# Evaluation of Severity and Factors Contributing to Foot Lesions in Endangered Ozark Hellbenders, *Cryptobranchus alleganiensis bishopi*

**DOI:** 10.3389/fvets.2020.00034

**Published:** 2020-02-04

**Authors:** Rebecca H. Hardman, Kelly J. Irwin, William B. Sutton, Debra L. Miller

**Affiliations:** ^1^Center for Wildlife Health, University of Tennessee, Knoxville, Knoxville, TN, United States; ^2^Arkansas Game and Fish Commission, Benton, AR, United States; ^3^Wildlife Ecology Laboratory, Department of Agricultural and Environmental Sciences, Tennessee State University, Nashville, TN, United States

**Keywords:** Hellbender, salamander, chytrid, *Bd*, amphibian disease

## Abstract

Arkansas populations of Ozark Hellbenders, *Cryptobranchus alleganiensis bishopi* have declined precipitously over the past few decades and are now limited to a single river. Biologists have also observed an increase of distal limb lesions with unidentified etiology and unknown role in morbidity and mortality of the species in this location. We documented lesions and collected associated individual size class data and pathogen samples in Ozark Hellbenders of Arkansas (*n* = 73) from 2011 to 2014 with the following two objectives: (1) document spatiotemporal patterns and severity of lesions present in this last remaining Arkansas Ozark Hellbender population, and (2) determine if host factors and infection status are associated with lesion severity. A scoring system was created from 0 to 7 based on lesion observations. Linear mixed model regressions followed by AICc model evaluation were used to determine associations among infection status for amphibian pathogens *Batrachochytrium dendrobatidis* (*Bd*) and Ranavirus as well as individual biometrics on lesion score. We discovered 93.2% of Hellbenders had lesions characterized by digit swelling that often progressed toward toe-tip ulceration. In severe cases we observed digital necrosis progressing to digit loss. Any recaptured individuals had the same or worse lesion score from previous captures. The top predictive model for lesion severity included individual mass and *Bd* infection status with a significant, positive association of *Bd* with increased lesion severity (β = 0.87 ± 0.39 S.E., C.I.: 0.11, 1.63). Our findings highlight a widespread and progressive disease that is an important factor to consider for the future of Ozark Hellbenders. This syndrome is presumptively multifactorial, and future studies will benefit from investigating several factors of host, infectious agents, and environment and their roles in disease manifestation for the purpose of developing effective, multi-faceted conservation strategies. A summary of potential etiologies and mechanisms is provided that may explain observed lesion distribution and that will be applicable to future disease and epidemiological investigations.

## Introduction

Human alteration of habitat has been linked to increased disease prevalence in many wildlife systems [see (1)]. Stream dwelling amphibians have been commonly used as biological indicators of environmental degradation due to their susceptibility to a variety of anthropogenic disturbances (2, 3). Specific consequences of degraded watersheds such as chemical runoff and increased sedimentation are known to negatively impact amphibian health [see (4, 5)].

Hellbenders (*Cryptobranchus alleganiensis*) are large, fully aquatic salamanders that inhabit cool, rocky, well-oxygenated rivers and streams in the eastern United States (6). The eastern subspecies (*C. a. alleganiensis*) inhabits a majority of the range with Ohio, Tennessee, and Mississippi River drainages extending from northeastern Mississippi to southern New York, and between southeastern Indiana and central West Virginia, with a disjunct population in central Missouri, while the Ozark Hellbender (*Cryptobranchus a. bishopi*) is a disjunct subspecies restricted to Ozark highland rivers of southern Missouri and northern Arkansas (7). Hellbenders may be particularly susceptible to increased sedimentation and other changes in water quality because they are fully aquatic and respire cutaneously (8). They require large cover rocks or crevices for breeding sites (6) and sediment-free interstitial spaces in gravel for larval and juvenile growth (9, 10).

Over the past few decades, population numbers have decreased appreciably in both subspecies. Many historical Hellbender populations are now extirpated or are functionally extinct (only large, senescent individuals remain) (11, 12). These drastic declines have resulted in federal listing of *C. a. bishopi* as an endangered subspecies (13) and inclusion of both *C. a. alleganiensis* and *C. a. bishopi* in appendix III of the Convention on International Trade in Endangered Species (CITES) of Wild Fauna (14). Ozark Hellbenders in Arkansas are believed to be extirpated from all but one river located in the northern portion of the state. Habitat degradation is hypothesized as the primary driver behind the declines of this subspecies. In one study in Missouri, decreased population densities of both subspecies were linked to decreased watershed buffers and associated change in stream parameters of temperature, conductivity, pH, and percent pebble (% of substrate in transect to be rocks of 4–64 mm diameter) (15). Moreover, Ozark Hellbenders in Arkansas (and populations in Missouri) have also been documented with distal limb lesions (16). These include feet with swollen, fused, or missing digits, and occasionally, exposed phalanges. A few cases are documented with partial to near complete foot loss. Similar but much less severe lesions have been observed in Eastern Hellbenders in Tennessee, but at lower frequency (17, 18).

Primary pathogens may also be negatively impacting *C. a. bishopi* populations and may contribute fully or in part to abnormalities, even if they are only part of a multifactorial process. Chytrid fungus (*Batrachochytrium dendrobatidis* or *Bd*) is present in *C. a. bishopi* (19, 20), and *C. a. alleganiensis* (21–23), but *Bd* prevalence is similar among both healthy and declining populations. However, in captive settings, *Bd* infection has resulted in mortality (24, 25), revealing its potential to cause disease in Hellbenders, especially in altered environments. The role of *Bd* in the morbidity and mortality of toe lesions remains unknown.

An emerging chytrid fungus, *Batrachochytrium salamandrivorans* (*Bsal*), has also been implicated in cases of skin lesions and mortalities in Europe. In addition, this pathogen appears to be more pathogenic to salamanders as opposed to frogs (26). Although *Bsal* has not been found in North America to date (21, 27), it is imperative to test salamanders with skin lesions to rule out a novel introduction.

Ranavirus is another emerging amphibian pathogen that is responsible for dramatic mortality events in captive colonies of the related Chinese Giant Salamander *Andrias davidianus* (28). Lesions from Ranavirus outbreaks in *A. davidianus* are identified by distal limb swelling, necrosis, and ulceration (29), which are similar to lesions observed in *C. a. bishopi*. Ranavirus has been found to infect wild Hellbenders, but without any obvious clinical signs at the time of sampling (18). However, Ranavirus infection in wild adult Hellbenders is negatively associated with calculated body condition score (BCS) (Hardman, unpublished data), highlighting the possibility that this virus may have unknown sublethal effects in Hellbenders.

Bacterial pathogens should also be considered, particularly opportunistic species associated with aquatic environments known to cause disease in immunosuppressed hosts. Two such species, *Citrobacter freundii* and *Aeromonas hydrophila* have been cultured from some, but not all lesions sampled in *C. a. bishopi* in Missouri (30). *C. freundii* is an opportunistic pathogen associated with aquatic ectotherms and is documented to produce a variety of sequelae including septicemic cutaneous ulcerative disease (SCUD) in turtles and necrotizing fasciitis in humans (31). *A. hydrophila* represents another opportunistic bacterium associated with mixed-pathogen infections that involves skin ulcerations and septicemia in both fish (32) and amphibians (33). However, when another study (34) applied culture-independent methods (16S amplicon sequencing) to identify bacteria from these lesions, they did not determine any consistent association between lesions and a particular bacterial species, further supporting this syndrome is likely of complex etiology.

Consequently, it remains unclear how host, pathogen, and environment may interact to produce a high prevalence of distal limb lesions. Further, no study has yet quantified lesion severity and applied an epidemiological evaluation of lesion presence. We documented lesions and collected associated individual size class data and pathogen samples in Ozark Hellbenders of Arkansas from 2011 to 2014 with the following two objectives: (1) document spatiotemporal patterns and severity of lesions present in this last remaining Arkansas Ozark Hellbender population, and (2) determine if host factors and infection status are associated with lesion severity.

## Materials and Methods

### Collection

Within Arkansas, Hellbenders are currently restricted to only one river, which we have chosen to not identify to prevent potential poaching. We performed 1 week intensive surveys each summer from 2011 to 2014. Surveys fell within a 3 week sampling window from late July to early August except for 2013 when high water levels prevented sampling, and sampling was delayed until September. These surveys were done before the onset of full breeding season which begins in early September. Disease sampling protocols were added to ongoing surveys for population monitoring. All sampling occurred under USFWS permit TE66039A-0 issued to KJI and Arkansas Game and Fish Commission. We sampled shallow and deep-water habitats (up to 4 m) using a hookah dive system (i.e., gasoline powered air compressor with tethered air supply lines to dive regulators). We also performed standard snorkeling to locate individuals in shallower water only. We also sampled Hellbenders via previously placed artificial nest boxes (35). Any animals encountered under cover objects or nest boxes were captured and placed in a clean soft cotton or mesh bag. Bags remained submerged in the river before and after animals were processed (University of Tennessee IACUC protocol # 2481-0916). We changed dive gloves between animal captures to reduce pathogen transfer and contamination of pathogen samples.

Standard measurements were recorded for total length (TL; cm), snout-vent length (SVL; cm), and mass (g). Captured animals were scanned for presence of a passive integrated transponder (PIT) tag, and number was recorded if present. New captures were marked subcutaneously with a PIT tag at the base of the tail as described in Hecht-Kardasz et al. (36), except without the use of post-injection adhesive. After processing, which took between 10 and 20 min per animal, all individuals were released at their point of capture. New containers, bags, and gloves were used between individuals. All equipment (including diving gloves) was cleaned with soap and soaked in 5% solution of either bleach or chlorhexidine for at least 10 min and rinsed thoroughly before use at another site. This protocol provides more than sufficient disinfection to prevent the spread of both Ranavirus (37) and *Bd* (38).

### Pathogen Sampling

Swabs for *Bd* quantitative PCR (qPCR) and tail tissue for Ranavirus qPCR were collected for another study evaluating disease prevalence (Hardman et al., unpublished data) and those results were incorporated into our lesion severity analysis. The methods for this were as follows:

To sample for chytrids (*Bd* and *Bsal*), we ran a sterile cotton swab (Fisherbrand product# 23400111) five times over each body region in the following order: dorsum, ventrum, inguinal areas, and all portions of the feet including toe-tips as described by Brem et al. (39). Although standard *Bd* swabbing protocol in frogs generally does not include the dorsum [see (39)], we included it because of unpublished reports of suspicious dorsal skin lesions associated with *Bd* infection in Hellbenders. We collected tissue samples for Ranavirus via a sterile punch biopsy (6 × 3 mm) or forceps and blade to remove a small tissue section of the dorsal tailfin near the tail-tip. We immediately placed swabs and tissue in sterile 1.5 mL microcentrifuge tubes with 70% ethanol for field storage and transport.

In 2012 we collected additional swabs exclusively from foot lesions in a subset of animals and sent these samples to Zoologix Inc. (Chatsworth CA) for additional targeted qPCR for *Citrobacter freundii* and *Aeromonas hydrophila*.

### Quantitative PCR (qPCR) of *Bd, Bsal*, and Ranavirus

Ethanol was removed via centrifugation under a fume hood to dry samples prior to extraction. We extracted DNA from swabs and tail tissue using a DNeasy Blood and Tissue Kit (Qiagen Inc., Valencia, CA, USA) and stored at −80°C. Separate qPCR singleplex assays were performed for each target pathogen (Ranavirus, *Bd, Bsal*) with the following 15 μL protocol: 7.5 μL 2X TaqMan™ Universal PCR Master Mix (Applied Biosystems™), 1 μL Forward Primer at 10 μM, 1 μL Reverse Primer at 10 μM, 0.5 μL Probe at 10 μM, 1 μL molecular grade water, and 4 μL sample DNA. Reaction temperatures were as follows for Ranavirus and *Bd*: 50°C for 2 min, 95°C for 10 min, and 45 cycles of the following: 95°C for 15 s and 60°C for 60 s. *Bsal* reaction temperatures were identical except with increased annealing temperatures of 62°C. We used primer and probe combinations published from Picco et al. (40) for Ranavirus qPCR reactions and Blooi et al. (41) for *Bd* and *Bsal* reactions. Each 15 μL reaction was performed in a 96-well plate via the QuantStudio™ 6 Flex Real-Time PCR System (Applied Biosystems) with a positive control from DNA extracted from pure culture for each pathogen and a water negative control. We performed separate singleplex assays for *Bd* and *Bsal* as opposed to the published duplex (41) assay to avoid potential inter-assay competition and subsequent false negatives (42). Each sample was run in duplicate and rerun when duplicates did not match within two cycle threshold (Ct) values. If a rerun continued to have a discrepancy, we removed the sample from our analysis. We determined animals to be positive for a pathogen with an average Ct value < 45.

### Lesion Scores

**Figure 1:**
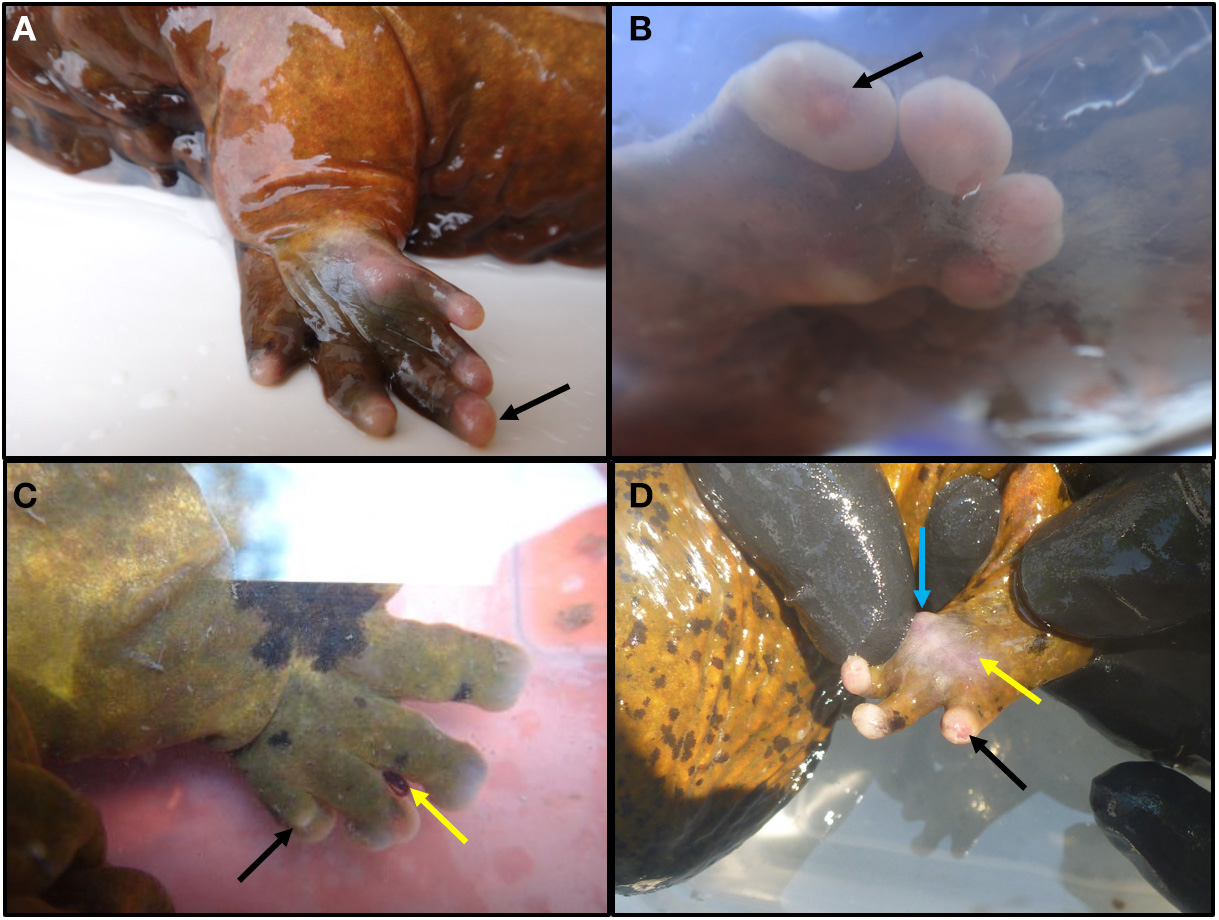
Examples of various degrees of lesions observed in Ozark Hellbenders *Cryptobranchus alleganiensis bishopi* in Arkansas. **(A)** Foot with all toes intact and slight erythema with digit III slightly swollen (black arrow). This clinical presentation equals a score of one if all four feet were in this condition. **(B)** Front foot with all swollen digits with a toe tip ulceration on digit IV (black arrow). This clinical presentation equals a score of three if all four feet were in this condition. **(C)** Toes have become flattened and are beginning to shorten. Digit IV is almost completely missing, and bone is beginning to protrude through the dorsal skin (black arrow). Note an attached leech in the interdigital space between digits II and III (yellow arrow). This clinical presentation equals a score of five if all four feet were in this condition. If any more toes were completely missing along with two or more foot pad ulcerations, a score of 6–7 would be given. **(D)** Digit IV is swollen and slightly erythematous with toe tip ulceration (black arrow). Skin on foot pad is thin, turned white gray, slightly transparent, and is close to ulcerating (yellow arrow). Underlying tissue is swollen and erythematous with an apparent nodule forming at the medial aspect (blue arrow). This clinical presentation equals a score of three if all four feet were in this condition.

Based on previous surveys we knew that lesions reported to be increasing in prevalence were generally restricted to distal limbs, and this is where we focused lesion quantification. Lesion scores were based on observations of chronic, non-healing, ulcerative areas of the feet and toes. We and others had noted several Ozark Hellbenders with various abnormal toes that were swollen, sometimes ulcerated, and in other cases semi-necrosed or missing. Some individuals had severe digit loss, while others had mild swelling. We wanted to quantify this difference as opposed to use a presence/absence approach to lesion prevalence. A lesion scoring system was further necessary because traumatic wounds to the feet and rest of body can occur from conspecific interactions during the breeding season. We wanted to prevent these healed scars and wounds from potentially confounding lesion assessment in Ozark Hellbenders by adhering to lesion score criteria that only evaluated non-healed lesions in the distal limbs (Table 1). Using a preprinted baseline drawing, individuals were recorded for lesion sub-type and anatomic location. Digits were noted as either “Healthy” (Figure 1A), “Bulbous” or “Swollen” (Figure 1B), “Shortened” (Figure 1C), or “Missing” (Figure 1C), with accompanying ulcerations on foot pads (Figure 1D), ulcerations on toe tips (Figures 1B,D), or completely missing limbs (picture not available). All drawings were created by the same field observer.

**Table 1:** Lesion scoring system used to quantify distal limb lesions in Ozark Hellbenders, *Cryptobranchus alleganiensis bishopi*.

**SCORE**	**No. foot pads ulcerated**	**No. toes missing**	**No. toes shortened**	**No. toes swollen**	**Description**
0	0	0	0	0	No observable distal limb lesions. Healed scars may be present. No active ulcerations or swelling.
1	0	0	0	1–5	1–5 swollen toes in one or two feet. No ulcerations on feet or toes. No shortened, fused, or missing toes
2	0	0	0	6–10	6–10 swollen toes affecting two feet. Toe tip ulcerations may be present. No shortened, fused, or missing toes
3	0–1	0	0	11–18	11–18 swollen toes affecting three or four feet. Toe tip ulcerations may be present. No shortened, fused, or missing toes. May have one footpad ulceration
4	0–1	0–1	1–5	1–17	1–5 shortened toes with 0–1 missing toes. Remaining toes swollen. May have one footpad ulceration
5	0–2	0–4	1–16	1–15	1–5 shortened toes with 2–4 missing toes OR 6+ shortened toes with zero missing toes. Remaining toes swollen. May have up to two footpad ulcerations.
6	0–2	5–18	1–13	1–13	5+ missing toes with up to two foot pad ulcerations
7	3–4	5–18	1–13	1–13	5+ missing toes with 3+ foot pad ulcerations.

*Scores are from 0–7. A score of 0 (blue) represents no distal limb lesions observed. Mild scores in green (1–2) have some swollen toes only, moderate scores in yellow (3–4) have most toes swollen and some shortened. Severe scores in red (5–7) denote several toes shortened or missing*.

A lesion scoring system with eight levels ranging from scores of 0–7 (Table 1) was created and scores were based on the number of toes affected, type of sub-lesion on each toe, and presence or absence of foot-pad ulcerations. A score of 0 denoted an individual with no digital lesions, whereas a score of 7 denoted an individual with very severe lesions in all four feet. These eight levels were determined based on natural breaks in lesion severity by number of toes swollen, progressing to number of toes shortened, and number missing. This allowed levels to account for differential amounts of toes affected by these three lesion subtypes without excessive levels to allow for quantitative analysis. Individuals receiving lesion scores of 1–2 primarily had toe swelling only. Scores 3–4 had most toes shortened and some missing. Scores 5–7 had several toes or even entire feet missing with remaining toes swollen or shortened. Score level cutoff and score designations to each animal were based on the standardized field drawings and were done blind to any other information of that animal.

### Lesion Severity Models

A linear mixed model was used to evaluate effects of infection status and individual factors on lesion severity. We originally chose the following fixed effect parameters: mass, TL, and presence or absence of *Bd* and Ranavirus. We created a third individual parameter of body condition score (BCS) by calculating residuals for each individual from residuals of the fitted polynomial plot of TL and mass. Total length was subsequently removed from our analysis because of its correlation with mass. Because of few detections of Ranavirus, it was excluded from our models. All models included a combination of our individual fixed effects listed and the following three random effects of: Individual ID, Year, and Site ID. Our model list is described in Table 2 which includes the following seven predictive models: Chytrid + Size, Chytrid + Condition, Chytrid only, Global, Size only, Condition only, Size + Condition, Random. All mixed models were created from the lme4 package (43) in RStudio (44). We evaluated relative model fit from AICc for all possible combinations of fixed effects to determine the top performing model via the AICcmodavg package (45) and considered models competitive when ΔAICc was < 2.0. Variables that appeared in more than one of the top models were model-averaged as recommended in Burnham and Anderson (46) to determine relative variable contribution in those models.

**Table 2:** List of Linear Mixed Models (LMM) included in AICc analysis in order of smallest to largest ΔAICc.

**Model name**	**Fixed effects**	**Random effects**	**K**	**LL**	**AICc**	**ΔAICc**	**AICcWt**
**Chytrid** **+** **Size**	***Bd*** **(0/1)** **+** **Mass**	**Indv ID, Site, Year**	**7**	**−137.25**	**290.22**	**0.00**	**0.26**
**Chytrid** **+** **Condition**	***Bd*** **(0/1)** **+** **BCS**	**Indv ID, Site, Year**	**7**	**−137.58**	**290.88**	**0.66**	**0.18**
**Chytrid only**	***Bd*** **(0/1)**	**Indv ID, Site, Year**	**6**	**−138.82**	**290.92**	**0.70**	**0.18**
**Global**	***Bd*** **(0/1)** **+** **BCS** **+** **Mass**	**Indv ID, Site, Year**	**8**	**−136.66**	**291.57**	**1.34**	**0.13**
Size only	Mass	Indv ID, Site, Year	6	−139.60	292.47	2.25	0.08
Condition only	BCS	Indv ID, Site, Year	6	−139.83	292.93	2.70	0.07
Size + Condition	Mass + BCS	Indv ID, Site, Year	7	−138.79	293.30	3.08	0.05
Random	—	Indv ID, Site, Year	5	−141.35	293.60	3.38	0.05

*Models evaluated individual and site factors on lesion severity (scored 0–7) of Ozark Hellbenders, Cryptobranchus alleganiensis bishopi in Arkansas. Fixed effects included presence/absence of chytrid fungus Batrachochytrium dendrobatids, Bd (0/1), mass (g), and body condition score (BCS). Random effects included Individual ID (Indv ID), site, and year. Top models of ΔAICc < 2.0 are noted in bold and were used for subsequent variable model averaging. Presence of Bd determined via qPCR from skin swab*.

## Results

### Pathogen Prevalence

Of the 73 individuals evaluated in this analysis, 23 (31.5%) were positive for *Bd, four* (5.5%) positive for Ranavirus, and 0% positive for *Bsal*. Two Ranavirus positive individuals were also infected with *Bd* (50.0%). For the subset of eight lesion-only swabs tested for opportunistic bacteria, 0% were positive for C. *freundii* and 0% positive for *A. hydrophila*.

### Lesion Severity Distribution

A total of 73 animals were assessed during this study with an average lesion score of 4.27 and a median score of five. Only five animals were considered lesion free (Lesion Score = 0; 6.8%), and all these five were negative for *Bd*. Lesion-free animals were generally smaller, falling within the ten smallest individuals captured (15th percentile in mass), weighing <300 g. Of the 12 recaptured individuals nine had progressively greater lesion scores from the previous recapture, whereas three maintained the same score (Table 3). We recaptured one individual twice, with an initial score of four in 2011, followed by a lesion score of six in both 2012 and 2014 (Table 3).

**Table 3:** Twelve recaptured individuals and subsequent change in lesion score between final and initial captures.

**Data ID**	**Recap no**.	**Final capture year**	**Final lesion score**	**Years between recap**	**Total score Δ**	**Score Δ per year**
151	1	2014	5	3	+0	+0.0
153	1	2015	7	4	+2	+0.5
158	1	2014	6	1	+2	+2.0
158	2	2014	6	3	+2	+0.7
161	1	2014	5	3	+0	+0.0
166	1	2012	4	3	+3	+1.0
168	1	2012	5	1	+0	+0.0
169	1	2014	7	1	+1	+1.0
177	1	2014	5	2	+4	+2.0
180	1	2015	4	1	+4	+4.0
190	1	2015	4	1	+0	+0.0
191	1	2015	7	1	+1	+1.0
Average			5.4	2	+1.6	+1.0

*Averages for scores listed at bottom. Note all lesion score changes are positive*.

### Linear Mixed Models and AICc

**Figure 2:**
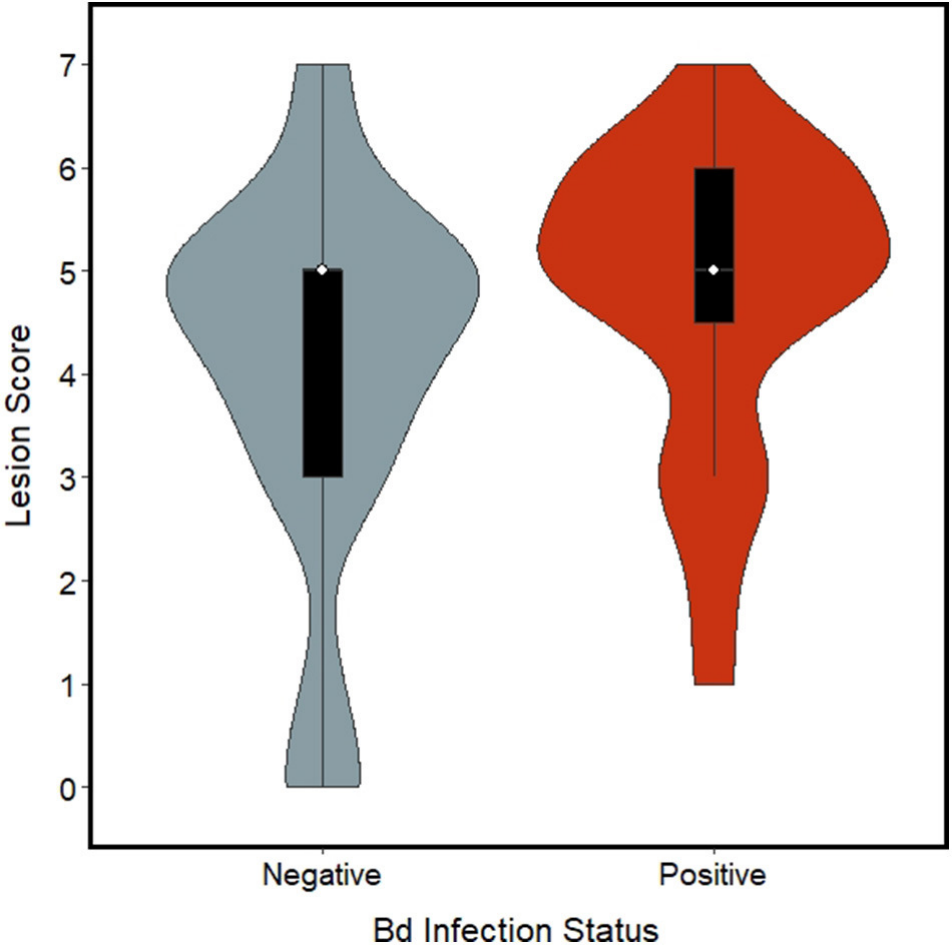
Violin plot with box plots embedded for lesion scores (ranging 0–7) from foot lesions of Ozark Hellbenders, grouped by *Batrachochytrium dendrobatidis* (*Bd*) infection status. *Bd* negative Hellbenders (blue-gray) had an average score of 4.06 whereas *Bd* positive Hellbenders (red) had an average score of 4.78.

Of the model sets evaluated, the Chytrid + Size, Chytrid + Condition, Chytrid only, and Global model sets were competitive based on AICc evaluation (Table 2). Of the model averaged coefficients from these model sets, *Bd* infection status was the only variable to have a significant effect on lesion severity (β = 0.87 ± 0.39 S.E., C.I.: 0.11, 1.63; Table 4). Lesions in *Bd*-positive animals had an average score of 4.78 in contrast to 4.06 in *Bd*- negative animals (Figure 2). Random effects also did not explain variation well with the following variances in the top model for Individual ID (0.6524 ± 0.8077 S.D.), Site (0.8336 ± 0.9130 S.D.), and Year (0.0 ± 0.0 S.D.). However, because site explained the most variation among random effects, we created a table showing average lesion score and pathogen prevalence by site location in order from most upstream to downstream (Table 5). This reveals no clear pattern with lesion score but does highlight two sites for further investigation which had the lowest average lesion scores of 2.3 and 1.5 for sites 8 and 13, respectively.

**Table 4:** Fixed effect variables in top models (ΔAICc < 2) listed with model-averaged results.

**Variable**	**β coeff**	**S.E**.	**95% C.I**.
***Bd*** **(0/1)**	**0.87**	**0.39**	**0.11, 1.63**
Mass	0.35	0.21	−0.05, 0.76
BCS	0.27	0.19	−0.11, 0.65

*Significant effects (based on 95% C.I.) noted in bold*.

**Table 5:** List of capture sites (exact location not disclosed) sampled for Ozark Hellbenders (*Cryptobranchus alleganiensis bishopi*) in Arkansas.

**Site order**	**Total captures**	**Ranavirus pos**.	***Bd* pos**.	***Bd* % pos**.	**Avg lesion score**
1	6	0	1	16.7	3.2
2	4	0	0	0.0	5.8
3	4	0	3	75.0	4.5
4	13	0	6	46.2	4.8
5	5	0	0	0.0	4.6
6	2	0	1	50.0	5.0
7	2	0	1	50.0	5.5
8	3	0	1	33.3	2.3
9	3	0	2	66.7	3.0
10	12	0	0	0.0	4.1
11	3	1	2	66.7	5.7
12	1	0	0	0.0	3.0
13	4	1	2	50.0	1.5
14	7	2	2	28.6	4.7
15	4	0	2	50.0	5.3
**Total**	**73**	**4**	**23**	**31.5**	**4.3**

*Site number represents relative location along stream from most upstream (Site 1) moving in order to most downstream (Site 15). Listed for each site are total captures, total positive samples for Ranavirus or Bd, and average lesion score. Total counts or averages across all sites shown in bottom row*.

## Discussion

This work presents novel data on the complexity and epidemiology of foot lesions in the remaining Arkansas Ozark Hellbender population. These lesions have only been mentioned to occur but have never been evaluated or fully described in published literature to date. Our results demonstrate these distal limb lesions are prevalent (93.2%) in *C. a. bishopi* in Arkansas. This is a larger percentage of affected animals than previously reported in Ozark Hellbenders in Missouri (49% in 2004 and 67–90% in 2006) (13). Lesions appear inflammatory in nature, although we do not have histological data to confirm presence of inflammation. They are non-healing and progressive, and we hypothesize that lesions start within soft tissues of the digits and then progress to the skin surface as grossly visible ulcerative lesions, followed by loss of tissue integrity, and necrosis. In recaptured individuals, we found that lesions are maintained or worsen in severity over several years and were always restricted to distal limbs.

Only five individuals from a total of 73 (6.8%) sampled were lesion free. These apparently healthy individuals were represented by some of the smallest individuals. Interestingly, our top predictive model included mass. Although this was not a significant effect in our model averaging analysis, mass was positively correlated with lesion severity. If this represents a true trend, it may reveal that older, larger individuals are more likely to have severe lesions. One interpretation of this phenomenon would be that lesions result from long term sequelae from a single historical event such as a chemical runoff or single pathogen outbreak, and younger individuals were simply not alive during the exposure period. A second interpretation is the etiological agent(s) that is/are responsible for these lesions require(s) long-term exposure to produce disease and younger Hellbenders are not old enough to have reached a certain critical exposure time for lesion development. Both hypotheses imply a chronic and progressive disease process which is supported by our recapture data showing individuals had either the same or worse lesion scores upon successive captures.

Our most important finding was that *Bd* infection status was in all top predictive models and represents the only statistically significant predictor to explain lesion severity. We confirmed a significant positive correlation of *Bd* infection with lesion severity. However, the causation behind this relationship remains unclear. Previous surveys of *Bd* in wild Hellbenders have been of little apparent value in population assessments, as *Bd* is present in this and several other Hellbender populations with no clear disease association (18, 21–23, 47). Still, *Bd* is an introduced pathogen to the United States, only to appear in Hellbenders after 1969 (20), and may be causing more damage to Hellbender health than understood. In captive Hellbenders, chytridiomycosis is considered a serious health threat, and both adults and juveniles have experienced mortality due to *Bd* (24, 25). Furthermore, mortality events appeared to follow times of stress such as with juvenile captive to wild translocations (25) where *Bd* may have been able to take advantage of weakened skin immunity. Although current literature does not support immunosuppression via chronic stress as a driver of classic *Bd* mortality events in amphibians (48), stress may play a role in *Bd* susceptibility of more tolerant species (49). Laboratory trials have shown that *Bd* infection intensity and incidence of chytridiomycosis can increase after alterations in host immunity via glucocorticoid administration in plethodontid salamanders (50) and Northern Leopard Frogs (51). Therefore, if wild *C. a. bishopi* are experiencing immunosuppression associated with lesion severity, *Bd* may have a greater chance of maintaining infection, and should still be considered a health threat in the wild.

Our data may underestimate the impact *Bd* has on *C. a. bishopi* because of our sampling time. In Crawfish Frogs, *Lithobates areolatus, Bd* infection prevalence and intensity has seasonal patterns where individuals often clear infection by late summer only to become reinfected in winter and spring (52). If *Bd* follows the same infection pattern in *C. a. bishopi*, it could be an important factor preventing wound healing and promoting progressive disease in cooler months when prevalence is much greater than when we sample in late summer. Still, we do not propose *Bd* as a primary or necessary etiologic agent for manifestation of these lesions, but stress that *Bd* may be important in progression of lesion severity through disruption of skin function and healing, especially in more severe cases.

We hypothesize that lesion formation occurs through a multifactorial process. Synergistic effects of introduced agents and altered or degraded habitat have been shown to produce disease in an otherwise resistant population. For instance, red tide blooms can affect sea turtle immune function and potentially drive increased prevalence of fibropapillomatosis (53). In amphibians, increased use of the herbicide atrazine can negatively affect health directly (54), but also indirectly though potentiating trematode infestations (55). Ozark Hellbenders in Arkansas are experiencing heavy sediment loads from agricultural land uses and subsequent decreased watershed buffers, which could result in concomitant increases of agricultural runoff (e.g., pesticides, herbicides, contaminants of poultry litter). All sites sampled for this study fall along one river with little forested buffer remaining and constantly eroding stream banks. We cannot ignore the fact that habitat degradation is likely contributing to the lesions in Ozark Hellbenders in Arkansas and may be causing chronic stress with long term consequences of immunosuppression. Further, in laboratory experiments of another salamander species, *Desmognathus ochrophaeus*, increased plasma corticosterone was associated with increased wound healing time (56), which supports a potential mechanism between chronic stress and lesions.

Ozark Hellbenders are likely immunocompromised from decreased genetic diversity. Low immunogenetic diversity is linked to increased disease incidence in other wildlife species e.g., malaria in great reed warblers (57), and facial tumors in Tasmanian Devils (58). *C a. bishopi* have lower MHC IIb diversity than their Eastern counter parts in Missouri (59) and combined with other factors, may have reached a threshold to become less resistant to one or more pathogens. Further, genetic bottlenecking could also increase frequency of rare alleles and chances for genetically driven disease. Rare alleles are hypothesized to play an important role in complex diseases in humans such as with autism (60) and warrants further investigation for Ozark Hellbenders.

Opportunistic bacteria are hypothesized to contribute to these digit and foot lesions once host immune systems are weakened, however, no specific opportunistic pathogen has yet been identified (30, 34). Our subset of lesion swabs was negative for both *C. freundii* and *A. hydrophila*, which lowers support for their role in lesion development. Several other pathogens including viruses and parasites still need to be considered. For instance, a case of progressive ulcerative dermatitis was reported in a captive treefrog, *Phyllomedusa bicolor*, associated with both viral and microsporidial systemic infection, showing potential for either to contribute to skin ulcerations (61).

Ranavirus should still be considered as a potential pathogen in producing these lesions despite having a low prevalence in our samples (5.5%). Lesions of ranavirosis in juveniles of related Chinese Giant Salamanders (*Andrias davidianus*) include inflammation, ulceration, and necrosis of the distal limbs (28, 29) that appear to be very similar to those observed in *C. a. bishopi*. Ranavirus infection in *C. a. bishopi* adults may result in sublethal disease with long term sequelae as opposed to dramatic mortality events observed in juvenile *A. davidianus*. If only a single Ranavirus infection event is needed to cause damage to begin lesion formation, and a typical Hellbender has a life expectancy of 20–30 years in the wild, probability of exposure and subsequent long-term sequelae may be fairly high. This concept could hold true for many other pathogens that cause damage to skin and vasculature, making the causative agent difficult to detect without long term monitoring.

It will be critical to understand the pathophysiology of this disease to determine the most effective course of action. Examples from domestic animals show us that complex diseases can be treated successfully if critical factors in disease manifestation are identified. For parvoviral disease in domestic dogs, the main etiologic agent is a virus (Parvoviridae), which cannot be targeted directly using antiviral drugs. Although disease can be prevented via vaccination, in unvaccinated dogs, mortality rates can be significantly reduced with antimicrobial treatment to prevent effects of secondary bacterial translocation of the gastrointestinal tract (62). Feline lower urinary tract disease (FLUTD) is a multifactorial process in domestic cats that can result in chronic urinary tract disease. Currently, the most effective treatment is not treating the animals, but rather treating the environment by removing sources of stress at home (63). Hellbender conservation managers should, therefore, focus attention not on a single factor but on an ecosystem. We may not be able to remove pathogens from these river systems, but we may be able to help reduce transmission between animals and reduce environmental stress. First, we can reduce artificially increased transmission and subsequent pathogen burden by adhering to aseptic sampling protocols in the field and in repatriation efforts. Second, we can focus efforts on habitat restoration. If bank restoration efforts decrease erosion and runoff, this may not only improve living and breeding space but simultaneously decrease disease incidence by reducing negative effects of environmental factors on host health.

This study documents a progressive disease affecting almost all sampled individuals in the remaining Ozark Hellbender population in Arkansas. The presentation of progressive lesions with a long differential list suggests that this disease is complex, and we highlight areas that need further research to fully understand factors in Ozark Hellbender health. Many questions still exist on the exact etiology of these lesions, and why they progress, but we do know that increased lesion severity is associated with *Bd* infection. We acknowledge that lack of histopathological data limits our inference into completely describing these lesions and recommend future studies to include histopathology to complement gross findings. Still, our lesion scoring system can be used to monitor changes in overall population health including response to restoration efforts. It can also be used to evaluate similar distal extremity lesions observed in other Ozark and Eastern Hellbender populations. Finally, Hellbenders are large, long-lived animals and may serve as sentinels for long term exposure to sublethal agents. The high prevalence of these lesions should not only motivate continued research in Hellbender health but should also raise concerns for potential health risks to other wildlife and human populations sharing these watersheds.

## Data Availability Statement

The datasets generated for this study are available on request to the corresponding author.

## Ethics Statement

The animal study was reviewed and approved by University of Tennessee IACUC.

## Author Contributions

RH, KI, and DM contributed to conception and design of the study. RH, KI, and WS completed filed sampling. RH and WS performed the statistical analysis. RH organized the database. RH wrote the first draft of the manuscript. All authors contributed to manuscript revision, read, and approved the submitted version.

### Conflict of Interest

The authors declare that the research was conducted in the absence of any commercial or financial relationships that could be construed as a potential conflict of interest.
